# Re-activation of Stem Cell Pathways for Pattern Restoration in Plant Wound Healing

**DOI:** 10.1016/j.cell.2019.04.015

**Published:** 2019-05-02

**Authors:** Petra Marhava, Lukas Hoermayer, Saiko Yoshida, Peter Marhavý, Eva Benková, Jiří Friml

**Affiliations:** 1 Institute of Science and Technology Austria, 3400 Klosterneuburg, Austria

## Abstract

Patterning in plants relies on oriented cell divisions and acquisition of specific cell identities. Plants regularly endure wounds caused by abiotic or biotic environmental stimuli and have developed extraordinary abilities to restore their tissues after injuries. Here, we provide insight into a mechanism of restorative patterning that repairs tissues after wounding. Laser-assisted elimination of different cells in *Arabidopsis* root combined with live-imaging tracking during vertical growth allowed analysis of the regeneration processes *in vivo*. Specifically, the cells adjacent to the inner side of the injury re-activated their stem cell transcriptional programs. They accelerated their progression through cell cycle, coordinately changed the cell division orientation, and ultimately acquired *de novo* the correct cell fates to replace missing cells. These observations highlight existence of unknown intercellular positional signaling and demonstrate the capability of specified cells to re-acquire stem cell programs as a crucial part of the plant-specific mechanism of wound healing.

## Highlights

• Restorative patterning replaces injured cells and allows plant tissues to heal
• The cells adjacent to the injury immediately induce oriented cell divisions
• Cell fate of divided cells is re-specified to correctly replace missing cells
• Restorative patterning involves activation of stem cell pathways in dividing cells

## Graphical Abstract

**Figure:**
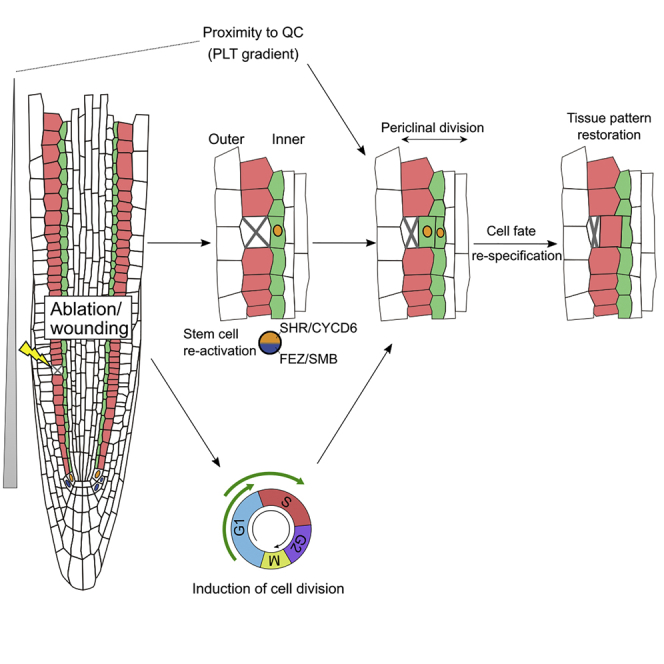

## Teaser

A process of restorative patterning in plant roots correctly replaces eliminated cells to heal local injuries despite the absence of cell migration, which underpins wound healing in animals.

## Introduction

Multicellular animals and plants emerged well after the split of these two lineages during evolution, and thus, these major eukaryotic groups utilize largely independent mechanisms to deal with challenges of multicellularity, such as cell-to-cell communication, development coordination, and tissue patterning. Unlike in animals, plant cells are encapsulated within rigid cell walls and thus cannot use cell migration during tissue patterning or wound healing. Therefore, plants rely mainly on strictly controlled orientation of cell divisions followed by the acquisition of specific cell fates (Rasmussen and Bellinger, 2018). The core cell-cycle machinery is conserved between animals and plants (Harashima et al., 2013); however, signals and mechanisms regulating the transition of cell-cycle stages and control of the cell division plane during patterning are presumably plant specific. Multiple molecular components and mechanisms of cell-fate specification have been elucidated in plants (Benfey et al., 1993, De Rybel et al., 2013), but little is known about how these individual mechanisms are activated and integrated during the concerted tissue-patterning processes.

Plants as sessile organisms have to regularly endure wounds caused by abiotic or biotic environmental factors; therefore, they evolved a remarkable ability to regenerate wounded tissues—e.g., reconnect interrupted vascular strands (Mazur et al., 2016) or regenerate whole complex structures, such as the root apical meristem (Efroni et al., 2016, Sena et al., 2009). It has been known for almost a century that harmed plant tissues activate cell division in adjacent cells and switch division planes to fill the wound with new daughter cells (Hartsema, 1926, Hush et al., 1990, Sinnott and Bloch, 1941). Later, when *Arabidopsis* root has been established as essential model for elucidation of patterning mechanisms in plants (Benfey et al., 1993, Dolan et al., 1993), more specific, microsurgical, laser-assisted cell eliminations allowed the observation of cell re-specification to regenerate lost cells—in particular, in the area of the root stem cell niche (van den Berg et al., 1995, Xu et al., 2006). Similar approaches also demonstrated that constant positional signaling is essential for maintaining the root meristem pattern during continuous development (Berger et al., 1998, Kidner et al., 2000). However, the phenomenon of wound healing and restoration of correct tissue pattern after injury has not been addressed specifically in *Arabidopsi*s roots. In particular, how the tissue re-acquires a correct pattern of cell types and what positional signaling mechanisms contribute to this remain unknown.

Here, we established a method of well-defined wounding by targeted cell elimination of individual cells or cell groups in *Arabidopsis* root meristem and combined this with extended live imaging at the vertical-stage microscope. This allowed analysis of the phenomenon of restorative patterning during wound healing. Restorative patterning involves activation of respective stem cell pathways and manifests in an immediate induction of cell division, controlled re-orientation of division planes, and acquisition of specific, correct cell fates. These observations provide insights into plant-specific wound healing and reveal previously unappreciated aspects of mechanisms underlying cell-division orientation, cell-fate acquisition, and positional signaling, as well as coordination of these processes during tissue patterning.

## Results

### Restorative Cell Divisions Induced by Local Wounding

**Figure 1:**
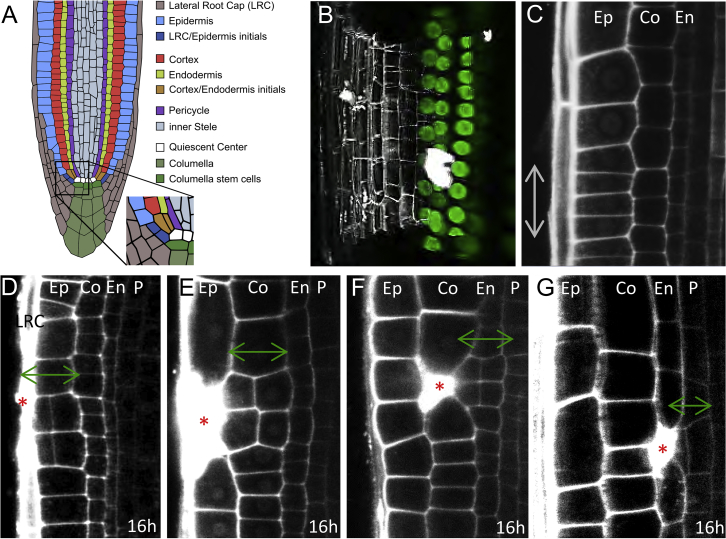
Ablation Triggers Restorative Cell Divisions in Inner Adjacent Cells (A) Cell types in the *Arabidopsis* root meristem. Inset shows magnification of stem cell niche. (B) 3D reconstruction of a single ablated endodermal cell in *SCR::SCR-YFP* root just after ablation. Propidium iodide (PI) stains cell walls and is not permeant to living cells and therefore was used to identify the ablated cell and intact neighboring cells. Shortly after ablation, PI becomes oversaturated and thus can partly overlap also with neighboring non-ablated cells. Later, PI staining is more restricted to the collapsed eliminated cell (see Figures S1C and S1D). (C) Anticlinal division of cells in root meristem (gray arrow). (D–G) Periclinal divisions of the inner adjacent cells (green arrows) after ablation. Ablation in LRC (D), epidermis (E), cortex (F), and endodermis (G) is shown. Total number of ablations: n = 30–60 per cell type. LRC, lateral root cap; Ep, epidermis; Co, cortex; En, endodermis; P, pericycle. Red asterisks: sites of ablation. See also Figure S1 and S7, Table S1, and Videos S1 and S2.

The root apex of *Arabidopsis thaliana* proved to be a great model for studying tissue patterning in plants. A small group of cells with stem cell-like properties surrounds the so-called quiescent center (QC) and generates all different cell types that form the root (Berger et al., 1998, De Rybel et al., 2016, Kidner et al., 2000, Kumpf and Nowack, 2015, Scheres et al., 2002). Once the different cell types are established by the stereotypic, asymmetric cell divisions, the daughter cells undergo only proliferative, anticlinal (perpendicular to the root axis) divisions that propagate the cell files on their way out of the meristem (Figures 1A and 1C).

We adapted the targeted UV-laser ablation technique (Marhavý et al., 2016, Xu et al., 2006) to eliminate specifically individual cells or cell groups in different cell layers of the root tip. 3D reconstruction of the area around the ablated cell confirmed an ablation of single cells with intact surrounding tissues (Figure 1B; Video S1).

Video S1. 3D Reconstruction of Ablated Cell in *SCR::SCR-YFP* Root Just after Ablation, Related to Figure 1

**Figure S1:**
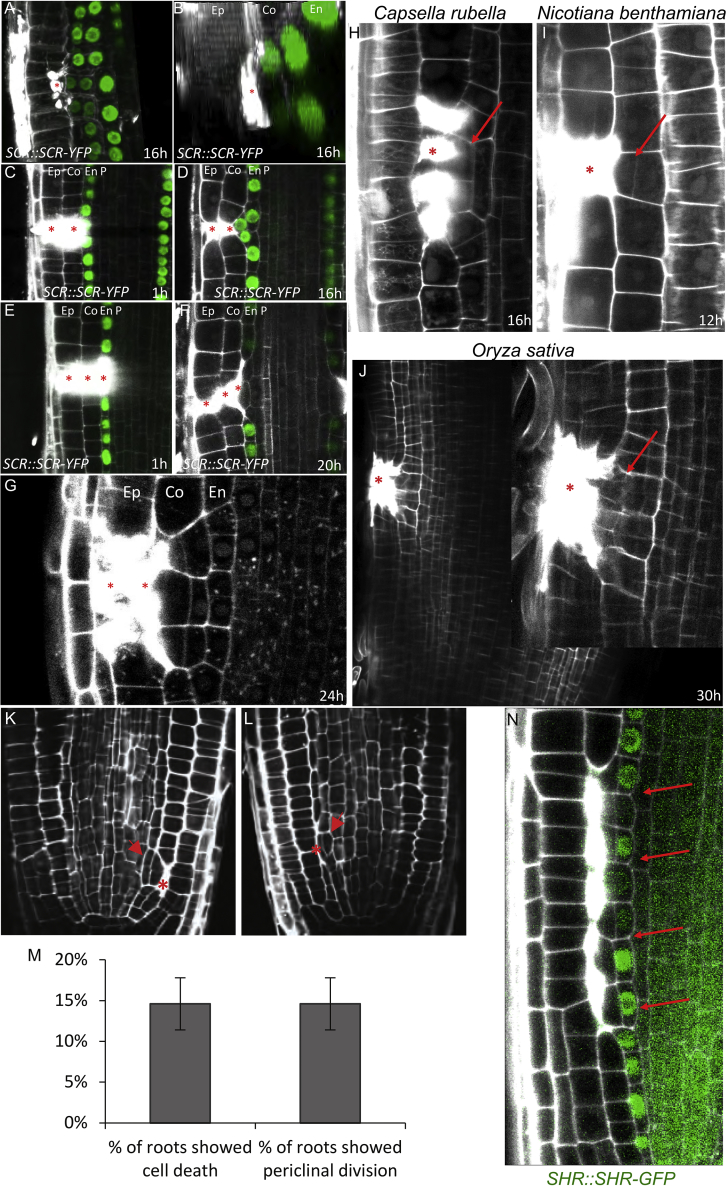
Effects of Non-targeted Cell Eliminations and Single/Multiple Ablations in *Arabidopsis* and Other Plant Species, Related to Figure 1 (A and B) 3D reconstruction front view (A) and top view (B) of a single ablated cortex cell followed by periclinal division of endodermal cells after 16 h. (C and D) Multiple ablations of epidermis and cortex after 1 h (C) and 16 h (D) of ablation. Total number of ablations; n = 10-20 per cell type. (E and F) Multiple ablations of epidermis, cortex and endodermis 1 h (E) and 20 h (F) after ablation. Total number of ablations; n = 10-20 per cell type. (G) Multiple ablations of epidermis and cortex lead to repeated divisions of endodermis cells to replace eliminated cells. (H-J) Single and multiple cell ablations of various cell types trigger periclinal cell divisions in inner adjacent cells in dicots *Capsella rubella* (H), *Nicotiana benthamiana* (I), and monocot *Oryza sativa* (J). (K and L) Restorative divisions in endodermis (K) and pericycle (L) after induction of non-targeted cell death by hydroxyurea. (M) Quantification of dead cells by hydroxyurea accompanied with periclinal divisions of neighboring cells. Data are represented as mean from four experiments ± SD (observed roots; n = 26, 15, 21, 41). (N) Restorative divisions in endodermis after natural death of cortex cells monitored in the *SHR::SHR-GFP* marker line. Ep: epidermis, Co: cortex, En: endodermis, P: pericycle. Red asterisks: ablated cells, red arrows: periclinal divisions. Roots were stained with propidium iodide.

The cell ablation led invariantly to a switch from anticlinal to periclinal (parallel to the root axis) division typically of the inner (very rarely also outer) adjacent cell, leading to eventual replacement of the eliminated cell (Figures 1D–1G; Table S1; Video S2). This capacity to initiate cell division was observed in all major cell types of the root meristem no matter in which layer the ablation was performed: ablation of a young lateral root cap (LRC) cell induced periclinal divisions in adjacent epidermis cells (Figure 1D), ablation of epidermis induced division of adjacent cortex (Figure 1E), ablation of a cortex cell led to the endodermis division (Figures 1F, S1A, and S1B; Video S2), ablation of endodermis induced pericycle division (Figure 1G). Periclinal cell divisions also occurred when larger injuries of two or three neighboring cells of different cell layers were ablated, always leading to the periclinal division of the first intact cell at the inner side of the eliminated cells (Figures S1C–S1F). In this case, the inner adjacent cells continued to divide periclinally until all dead cells were replaced (Figure S1G). To eliminate cells by a different method, we used treatment with hydroxyurea (HU), an inhibitor of ribonucleotide reductase, which inhibits DNA replication and induces cell death (Cools et al., 2011). We observed random cell deaths in cortex and endodermis, and all these events led to periclinal divisions of the inner adjacent cells (Figures S1K–S1M), as seen in the case of laser-assisted cell elimination. Naturally occurring wounds occasionally observed even in laboratory growth conditions induced similar periclinal divisions in inner adjacent cells (Figure S1N).

Video S2. 3D Reconstruction of Ablated Cortex Cells after 16 h, Related to Figure 1

Moreover, when we ablated cells in the roots of dicots *Capsella rubella*, *Nicotiana benthamiana*, and the monocot *Oryza sativa*, we observed the same phenomenon of wound-induced activation of periclinal cell division (Figures S1H–S1J). Genetics and marker lines analysis (Figures S7A–S7G) revealed that the injury-induced division does not require known regulators of periclinal cell division (LHW, TMO5, LOG4, TCS), mechanosensing (FERONIA, THESEUS, MCA1 and MCA2, MSLs), or formation of the preprophase band (*trm6,7,8*), suggesting existence of unknown signaling mechanism conserved across higher plants.

These observations show that controlled, localized injuries in the root meristem of various species invariantly induce periclinal cell divisions in the inner adjacent cells. Notably, in the undisturbed situation, cells in these root regions undergo only anticlinal divisions, but the wound does not, typically, heal by this type of simple proliferation of the injured cell file. Instead, it requires signaling to the different cell files adjacent to the inner side of the wound, possible activation of their division, and the re-orientation of the division plane. As these processes ultimately lead to the regeneration of the wounded tissue, we termed this previously unappreciated phenomenon “the restorative cell division.”

### Competence to Initiate Restorative Cell Divisions

**Figure 2:**
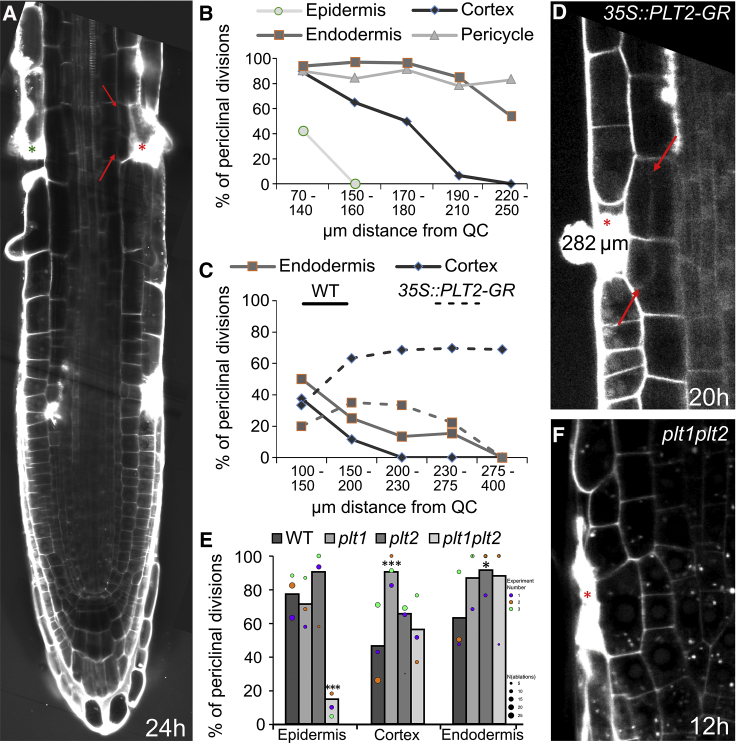
Decreasing Competence of Restorative Divisions Correlates with PLT Activity (A) Tile scan of a root 24 h after ablation of cortex and epidermis. While ablation of cortex cells (red asterisk) induced periclinal cell divisions in endodermis (red arrows), ablation of epidermis (green asterisk) did not induce periclinal division in cortex, which lost the competence to divide. In contrast, ablation of epidermis closer to the quiescent center induced the periclinal cortex cell divisions. Marked ablated cells moved from meristem into elongation zone during growth. (B) Number of periclinal divisions decreases with distance from QC as measured in various cell types after 16 h depending on distance from the QC at the time of ablation (μm). Total number of ablations: n = 15–50 for every 10 μm (70–250 μm). (C) Number of periclinal divisions in cortex and endodermis cells depending on the distance from the QC at the time of ablation is increased after PLT2 induction as measured in WT and *35S::PLT2-GR* (treated with 5 μM DEX 1 h prior ablation) 12 h after ablation. Roots with ectopic oblique divisions were excluded from the quantifications. Number of ablations per data point: n = 3–26. (D) Overexpression of PLT2 starting 1 h before ablation triggered periclinal and oblique divisions in inner adjacent cells. Cortex cells are already partly elongated. Ablation was performed at 282 μm from the QC. (E) Number of periclinal divisions after ablation in *plt1*, *plt2* single, and *plt1plt2* double mutants in different cell layers at random distance from the QC after 12 h. Data are represented as weighted mean (bar) and individual experiments (dots, area indicates sample size). Asterisks correspond to p values from conditional logistic regression (CLR); *plt1* (cortex): 0.000538, *plt2* (endodermis): 0.0231, *plt1plt2* (epidermis): 9.99E−07. (F) Periclinal division was not induced in epidermis in the *plt1plt2* double mutant 12 h after ablation. Red asterisks: sites of ablation. Roots were stained with PI. See also Figure S2 and Table S1.

**Figure S2:**
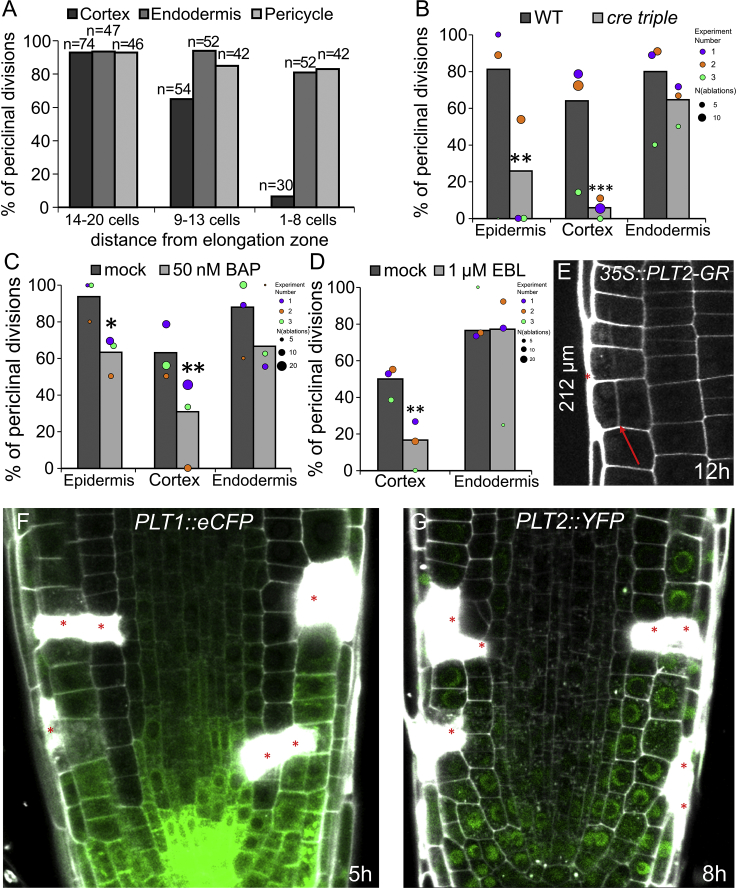
Role of Meristem Activity and PLT Expression Gradient in Regenerative Competence, Related to Figure 2 (A) Rate of periclinal divisions depends on the distance from the meristem end to ablated meristematic cell (total number of ablations is indicated in chart). (B-D) Rate of periclinal divisions 12 h after ablation in *cre1 ahk2 ahk3* triple mutants **(B),** in seedlings treated with 50 nM cytokinin (benzyl amino purine, BAP) (C) and with 1 μM 24-epibrassinolide (EBL) (D). Data are represented as weighted mean (bar) and individual experiments (dots, area indicates sample size); asterisks indicate p values from CLR. (E) Regeneration of epidermis competence can be greatly increased by overexpression of PLT2 as LRC ablated at 212 μm distance from the QC was able to trigger periclinal cell division in epidermis 12 h after ablation. (F-G) Expression patterns of *PLT1::eCFP* (F) and *PLT2::YFP* (G) were not affected by ablation of different cell types in cells undergoing restorative divisions. Red asterisks: sites of ablation, red arrows: periclinal divisions. Roots were stained with propidium iodide.

Our observations suggested that various root cell types have a capacity to initiate periclinal cell divisions in response to wounding also well outside of the stem cell niche, to where this type of divisions is normally confined. Thus, we tested systematically the competence of different cell types depending on their distance from the stem cell niche. We eliminated cells at different positions in the root apex (Figure 2A) and measured their position within the root apex by different means at 1 h after cell ablation along with the ability of inducing restorative cell divisions after 16 h (Figures 2B and S2A). We found that the capacity of cells to induce restorative divisions gradually decreases at different rates in various cell types with the distance from the QC (Figure 2B). It appears that epidermis loses the responsiveness to cell ablation first (around 150 μm), followed by cortex (190 μm) and endodermis (220 μm), while pericycle maintains its competence to initiate restorative divisions—consistent with its behavior as a “dormant meristem” throughout the whole root development (Marhavý et al., 2016).

This demonstrates that epidermis, cortex, endodermis, and pericycle cells all are competent to initiate restorative cell divisions well outside of the stem cell niche with their competence decreasing at different rates with the distance from it.

### PLETHORA Regulators Mediate Competence for Restorative Cell Divisions

To get further insight into the mechanism underlying decreasing competence to initiate restorative cell divisions in more differentiated parts of the root meristem, we analyzed roots with shortened meristems. First, we analyzed *cre1 ahk2 ahk3* triple mutant of the cytokinin receptor genes and plants treated with cytokinin and brassinolide, which all displayed reduced meristematic activity (Higuchi et al., 2004, Marhavý et al., 2011, Zhu et al., 2013). While the ability to induce restorative cell division in epidermis and cortex was significantly reduced, division rate in endodermis was only slightly decreased (Figures S2B–S2D). Thus, as expected, the regenerative competence in some cell types depends highly on meristematic activity.

Next, we analyzed the potential involvement of members of the PLETHORA (PLT) regulators of stem cell activities, which form an expression gradient decreasing with distance from the QC (Galinha et al., 2007; Figures S2F and S2G), thus correlating with the decrease of regenerative competence. Moreover, ectopic expression of PLT1 and PLT2 initiates a variety of phenotypes, including increased meristem size and ectopic root formation throughout the plant (Aida et al., 2004, Galinha et al., 2007). Thus, we analyzed regeneration competence 12 h after ablation in the transition and elongation zones of the *35S::PLT2-GR* line. Ectopic PLT2 expression strongly rescued the capacity of cortex cells to induce restorative divisions after epidermis ablation even when they already left the division zone of the root tip (Figure 2C); also, in some cases, ablation of LRC cells at the end of the division zone induced periclinal or oblique divisions in epidermis (Figure S2E). Similarly, the cells of endodermis slightly regained the competence to divide periclinally following cortex ablations even outside of the division zone (Figure 2C). Notably, induction of PLT2 expression as short as 1 h prior to ablation was sufficient to increase the rate of restorative divisions within (150–200 μm) and at the end of the division zone (200–230 µm) and even allowed cells that already left the division zone to induce restorative divisions (at 275–400 μm from 0% in wild type [WT] to ∼70% in *35S::PLT-GR*; Figure 2D).

In a reciprocal experiment, we analyzed *plt1* and *plt2* mutants and found that single mutants showed no reduction in periclinal division rates in epidermis but possessed increased number of periclinal divisions in cortex (*plt1*) and endodermis (*plt2*). Notably, *plt1plt2* double mutant is strongly defective in restorative divisions in epidermis (Figures 2E and 2F).

Our results identified PLT transcription factors as being necessary and sufficient components of the root cell competence to initiate restorative division. Moreover, the regenerative competence depends on PLT expression levels and correlates with the PLT expression gradient in the root.

### Accelerated Progression through Cell Cycle during Restorative Division

**Figure 3:**
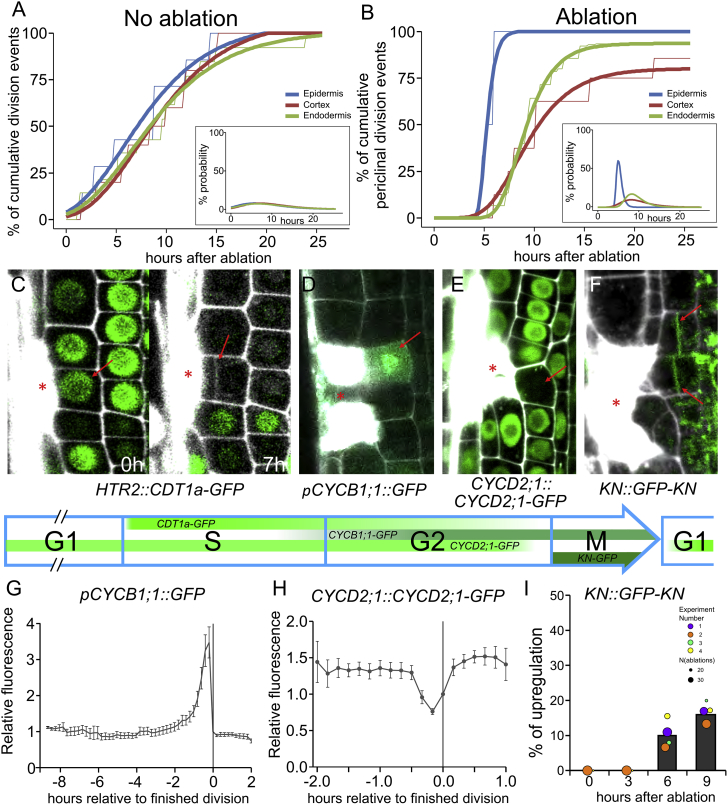
Accelerated Activation of Cell-Cycle Progression (A and B) Cumulative divisions in roots monitored by vertical-stage microscopy (observed roots; n = 10 for each experiment). Without ablations, all cell types divided slowly at the same rate, as seen in the quantification of anticlinal divisions in roots with virtual ablations (n = 4 per root) (A), whereas division rates were much faster and differed markedly between cell types after wounding, as seen in quantifications of periclinal divisions in roots with laser ablations (n = 3 per root) (B). Thin lines show raw data, and thick lines show fitted curves calculated by least-square estimates (sigmoidal curves: y = a^∗^eˆ(−b^∗^eˆ(−c^∗^x))). Insets show derivatives of fitted curves. (C–F) Expression pattern of cell-cycle regulators during restorative divisions. Inner adjacent cells of ablated cells were already in S phase during ablation as indicated by strong *HTR2::CDT1a-GFP* signal (left) but still performed periclinal cell division (right) (C). Mean *CDT1a-GFP* fluorescence intensity at 0 h after ablation relative to the first time point after ablation: 9.3 ± 1.6 (n = 8 cells). G2/M transition marker *pCYCB1;1::GFP* is specifically upregulated before restorative divisions (D), while *CYCD2;1::CYCD2;1-GFP* leaves the nucleus shortly before division (E), and cytokinesis marker *KN::GFP-KN* is upregulated at the newly formed cell plate (F). Progression of cell-cycle phases are shown in blue arrow with green stripes indicating expression patterns as observed or previously reported (for *CDT1a-GFP*, see Yin et al., 2014). (G–I) Quantification of fluorescent signals of marker gene expressions of *pCYCB1;1::GFP* (G), *CYCD2;1::CYCD2;1-GFP* (H), and *KN::GFP-KN* (I) during restorative divisions after ablation. Data for *pCYCB1;1::GFP* and *CYCD2;1::CYCD2;1-GFP* were collected by long-term vertical-stage imaging over 24 h and are presented as mean fluorescence intensity relative to time point of finished division of subsequently followed cells (n = 16 and n = 10, respectively) ± SEM and is supported by three qualitative experiments. *KN::GFP-KN* is represented as mean (bar) and individual experiments (dots, area indicates sample size) of qualitative analysis of GFP signals. Red asterisks: sites of ablation. Roots were stained with PI. See also Figure S3, Table S1, and Video S3. Video S3. Long-Term Imaging of Cell Ablations and Restorative Divisions by Vertical-Stage Microscopy, Related to Figure 3

**Figure S3:**
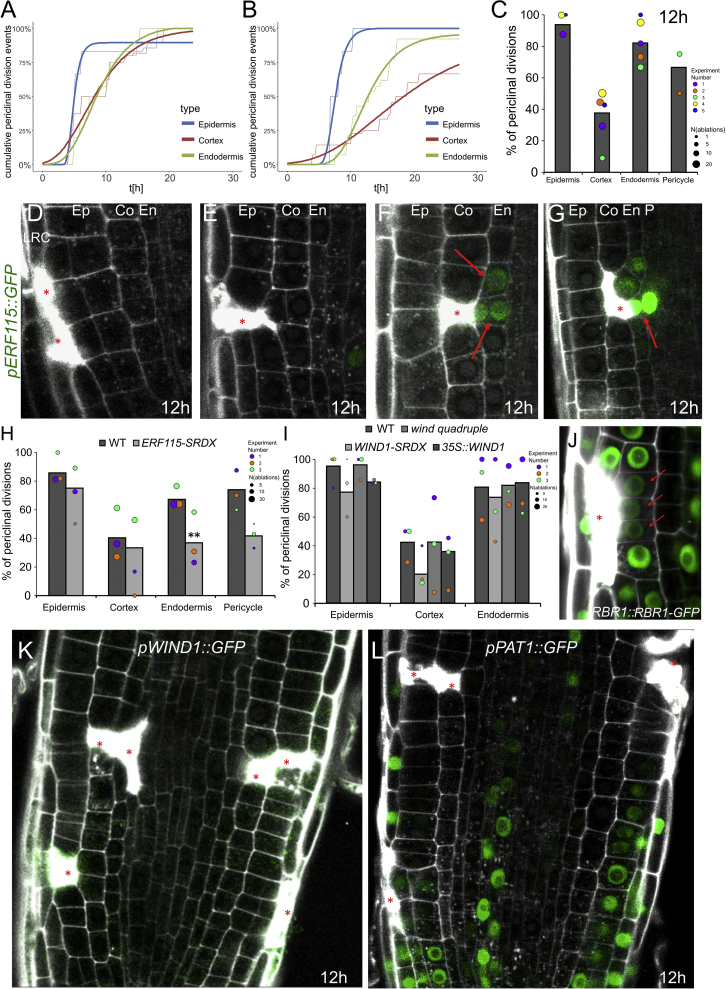
Activation of Cell-Cycle Machinery Triggered by Ablation, Related to Figure 3 (A and B) Restorative division rate in cortex cells varies highly between different experiments: compare red line in (A), (B) and Figure 3B. Cumulative periclinal division events in 10 roots with 1 ablation site per cell type each were monitored by vertical stage microscopy. Thin lines show raw data and thick lines show fitted curves calculated by least square estimates (sigmoidal curves: y = a^∗^eˆ(-b^∗^eˆ(-c^∗^x))). (C) Restorative division rates differ highly between cell types 12 h after ablation. Data are represented as weighted mean (bar) and individual experiments (dots, area indicates sample size). (D-G) *ERF115::GFP* expression after ablation of LRC (D), epidermis (E), cortex (F) and cortex and endodermis (G). Upregulation is specific to restorative divisions in endodermis and pericycle. (H) Rate of periclinal divisions after ablation in *ERF115-SRDX* line. Data are represented as weighted mean (bar) and individual experiments (dots, area indicates sample size); p values from CLR: epidermis: 0.178, cortex: 0.134, endodermis: 0.00232, pericycle: 0.129. (I) Rate of periclinal divisions induced by ablation in *wind* quadruple mutants, *WIND1-SRDX* line and *35S::WIND1* line in various cells. Data are represented as weighted mean (bar) and individual experiments (dots, area indicates sample size). (J) Specific downregulation of the G1/S phase transition inhibitor *RBR1::RBR1-GFP* was only observed in rare cases in inner adjacent cells prior to restorative divisions. (K) Expression of *pWIND1::GFP* was not detected in roots after 12 h of ablation, during restorative division initiation. (L) Expression of *pPAT1::GFP* was not upregulated after 12 h of ablation, during restorative divisions. Red asterisks: sites of ablation. Roots were stained with propidium iodide.

To analyze whether the wounding only changes the plane of cell division from anticlinal to periclinal or, in addition, also accelerates the entry into and/or progression of the cell cycle, we performed a time series counting the divisions associated with the ablation events using the vertical-stage microscope with automatic tracking (von Wangenheim et al., 2017). Roots without ablations underwent anticlinal divisions in a spatially and temporarily regular manner, which can be observed as a nearly linear increase of cumulative divisions over time. Fitted sigmoidal curves showed nearly flat derivatives, showing that there is equal probability of cell divisions during the observed time frame in all cell types (Figure 3A). In contrast, wounding-induced periclinal divisions showed different but very uniform characteristics within different roots (Video S3). After a lag time of ∼5–7 h, the cumulative periclinal division events from ∼40 ablation sites increased rapidly and reached a maximum within 10 additional h. The derivatives of the fitted sigmoidal curves showed that ablation highly increased the probability of divisions (Figure 3B). Additionally, the speed of induction of restorative cell divisions varied markedly between cell types. Whereas epidermis cells induced divisions almost uniformly, endodermis division events spread over a slightly broader timescale. The ability to specifically induce divisions in cortex cells varied from a rate comparable to endodermis (Figure S3A) to situations without wounding (Figure S3B) but was mostly somewhere between these two extrema (Figure 3B). In most cases for cortex (60%) and epidermis (70%), exponential curves showed a better fit, while endodermis curves in majority (70%) fitted better to a sigmoidal behavior. Pericycle cells are not efficiently trackable for prolonged periods, but endpoint (e.g., 12 h) measurements after ablation revealed induction times similar to the endodermis (Figure S3C). Despite these differences, the minimal observed induction time of ∼5 h is consistent within all cell types.

Next, we observed progression of the cell cycle after ablation and analyzed which transition steps are required for the initiation of restorative cell divisions. We tested the expression of S-, G2/M-, and cytokinesis-specific markers, which also showed dynamic changes in proliferative cell divisions occurring in a seemingly random diverse spatial and temporal distribution within the root meristem (Dewitte and Murray, 2003). The evolutionary conserved inhibitor of the G1-to-S-phase transition, RETINOBLASTOMA-RELATED1 (RBR1) (Ebel et al., 2004), in the *RBR1::RBR1-GFP* marker line showed occasionally specific downregulation in the inner neighbor cells to the wound before restorative divisions were induced (Figure S3J). However, in long-term imaging experiments, we could not observe any specific expression changes in most cells that perform restorative divisions. So we investigated a synthetic S-phase marker, *HTR2::CDT1a-GFP*, which gets promptly activated when a cell enters the S phase and gets slowly degraded until the cytokinesis (Yin et al., 2014). Most cells in the root meristem showed strong expression in this marker, meaning they were currently in S phase. When we ablated cells adjacent to such cells, we observed that S-phase cells were able to trigger restorative divisions, including the switch to periclinal orientation (Figure 3C), suggesting that a G1-to-S-phase transition (e.g., by downregulation of RBR1) is not crucial for restorative divisions.

A transcriptional activator of G1-/S-phase transition genes, ETHYLEN RESPONSE FACTOR 115 (in the *ERF115::GFP-NLS* line) was upregulated around 4–5 h after ablation, prior to restorative cell divisions as described previously (Heyman et al., 2016, Heyman et al., 2013). Nonetheless, the ERF115 upregulation is specific to endodermal and stele cells next to wound and was not observed in epidermis and cortex cells (Figures S3D–S3G). Accordingly, the *ERF115-SRDX* dominant-negative line showed a significantly reduced number of restorative cell divisions in endodermis and a lower number of divisions in pericycle (Figure S3H). This suggests that, although not required, the transition from G1 to S phase is part of the restorative division initiation and increases the amount of restorative cell divisions that occur within 12 h. The binding partner of ERF115, PHYTOCHROME A SIGNAL TRANSDUCTION1 (PAT1; Heyman et al., 2016), and one of the downstream targets, WOUND INDUCED DEDIFFERENTIATION1 (WIND1, Iwase et al., 2011), did not show any specific expression changes during restorative divisions (Figures S3K and S3L). Additionally, *wind1* mutant and overexpression lines did not show any defects in periclinal division after ablation (Figure S3I).

Next, we observed the progression through the G2 phase and its importance for restorative divisions. Using long-term imaging during restorative divisions, we observed upregulation of the G2/M marker *pCYCB1;1::GFP* (Ubeda-Tomás et al., 2009) and downregulation of *CYCD2;1::CYCD2;1-GFP* (Sanz et al., 2011) as it normally occurs during progression through G2 phase (Figures 3D–3E and 3G–3H). Additionally, we observed the cytokinesis onset and progression as marked by the cytokinesis-specific protein KNOLLE in *KN::GFP-KN* (Reichardt et al., 2007) at the newly formed cell plate during restorative divisions (Figures 3F and 3I). This demonstrates that enhanced progression through the cell cycle requires the differential expression of canonical G2- and M-phase genes.

Thus, the quantifications of cumulative cell divisions revealed that wounding-induced restorative divisions occur significantly faster than regular proliferative divisions. Notably, whereas for the normal, proliferative cell division rates, there are no visible differences between the cell types, the restorative cell divisions show strongly divergent rates of divisions in different cell types. Whereas these quantifications cannot discriminate between accelerated entry and accelerated progression of the cell cycle, the molecular markers further confirmed the accelerated cell cycle progression. Additionally, it showed that coordinated G1-/S-phase transition is not required for restorative cell division, while G2-/M-phase transitions are tightly controlled after wounding.

### Restorative Cell Divisions Generate Daughter Cells with Distinct, Correct Fates

Next, we addressed the cell fate of the daughter cells generated by restorative cell divisions. In the root, the stereotypic divisions of the stem cells generate all cell types, which, subsequently after leaving the stem cell niche, have their fixed identities propagated by the proliferative anticlinal cell divisions (van den Berg et al., 1995, Berger et al., 1998, Dolan et al., 1993, Kidner et al., 2000).

**Figure 4:**
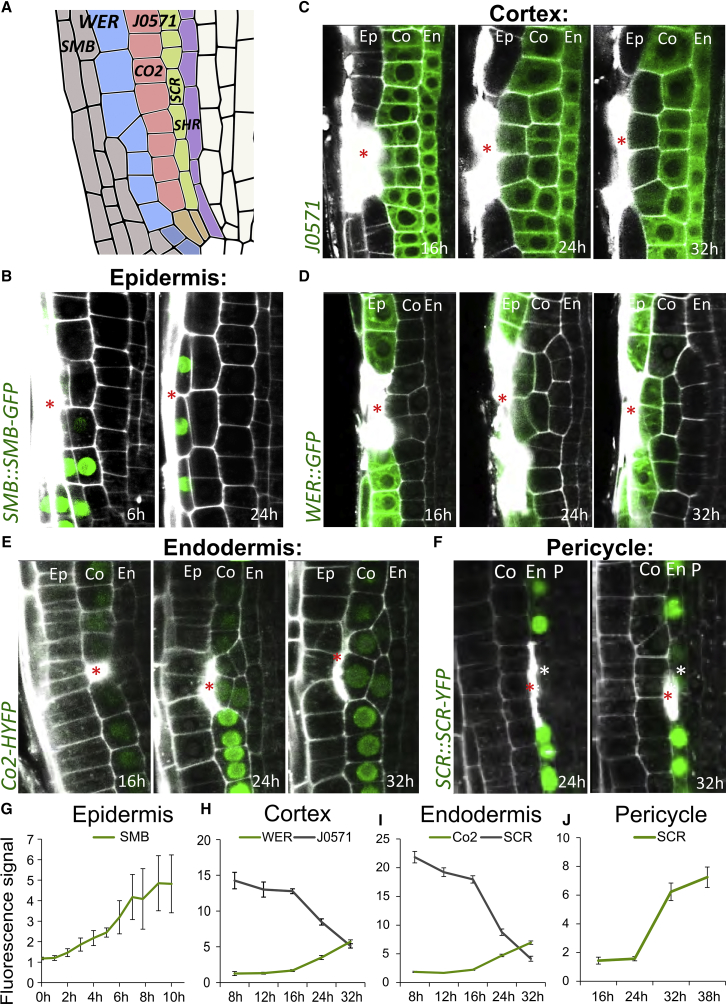
Restorative Cell Divisions Generate Daughter Cells with Distinct Cell Fates (A) Expression domains of marker lines in *Arabidopsis* root meristem. (B) Ablation of LRC cells. Expression of LRC marker *SMB::SMB-GFP* is restored in newly formed LRC cells. Total number of ablations: n = 5 for all time points. (C and D) Ablation of epidermal cells. Expression of cortex and endodermis marker *J0571* is downregulated in the outer daughter cells of periclinally divided cortex cells (C), while epidermis marker *WER::GFP* is upregulated (D). Numbers of ablations per time point: n = 19–77 (*J0571*), n = 16–66 (*WER::GFP*). (E) Ablation of cortex. Expression of cortex marker *Co2::HYFP* is upregulated in outer daughter cells of periclinally divided endodermis cells, while endodermis marker *SCR::SCR-YFP* is downregulated (see Figure S4A). *Co2::HYFP*; n = 43–79 ablations per time point. (F) Ablation of endodermis. Expression of *SCR::SCR-YFP* is upregulated in outer cells of periclinally divided pericycle cells. n = 13–23 ablations per time point. (G–J) Quantification of fluorescent signals of marker gene expressions in periclinally divided cells after ablation. Relative signal intensity between outer/inner daughter cells in periclinally divided epidermis (G) and average fluorescent signal in cortex (H), endodermis (I), and pericycle (J) at different time points. Outer daughters were measured if the inner adjacent cells divided multiple times (as seen in D). Data are represented as mean ± SEM. Red asterisks: sites of ablation. Roots were stained with PI. See also Figure S4 and Table S1.

To assess possible cell-fate changes, we used specific marker lines for different cell types (Figure 4A) and followed their expression during and after restorative cell divisions. After ablation of LRC cells in the LRC-specific marker line *SMB::SMB-GFP* (Willemsen et al., 2008), we measured GFP fluorescence in both daughter cells generated by a restorative division of the epidermis. The SOMBRERO (SMB)-GFP expression in outer daughter cells, facing the ablation site, as compared to the inner cell, became significantly higher 4 h after division (Figures 4B and 4G). This indicates that the outer epidermis daughter cell generated by the restorative division rapidly acquired the LRC fate replacing the eliminated cell.

Next, we observed dividing cortex cells after ablation of epidermal cells in the roots of the cortex and endodermis-specific marker line *J0571* (Haseloff, 1999, Mylona et al., 2002). The difference between inner and outer daughter cells appeared about 24 h after ablation, and the GFP signal almost disappeared from the outer cells after 32 h, indicating that these cells were losing cortex cell fate and acquired their new fate (Figures 4C and 4H). Additionally, at about 24 h after epidermis ablation, outer cells of periclinally divided cortex cells started to express the epidermal marker *WER::GFP* (Lee and Schiefelbein, 1999), confirming that these cells were in the process of acquiring the cell fate of the eliminated epidermal cell (Figures 4D and 4H).

**Figure S4:**
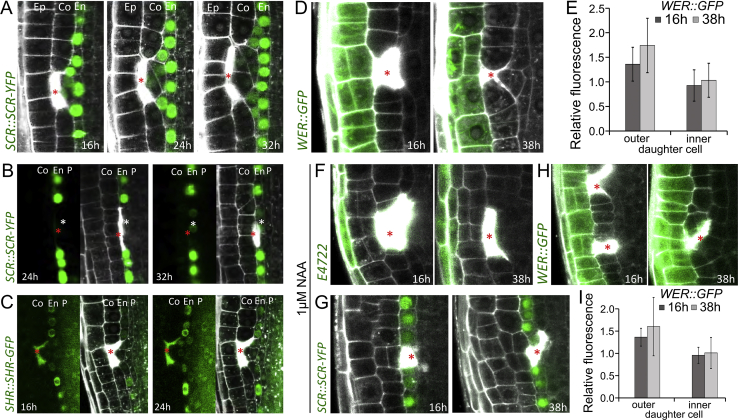
Cell Fates of Inner and Outer Adjacent Cells of Ablation, Related to Figure 4 (A) Ablation of a cortex cell results in decreased expression of endodermis marker *SCR::SCR-YFP*. Total number of ablations; n = 42 (8 h), n = 47 (12 h), n = 95 (16 h), n = 63 (24 h), n = 47 (32 h) - see increased cortex marker *Co2::HYFP* expression in Figure 4E. (B and C) Ablation of endodermal cell results in upregulation of *SCR::SCR-YFP* in pericycle (B). Ablation of cortex and endodermis results in nuclear localization of *SHR::SHR-GFP* (C) in outer daughter cells of periclinally divided pericycle cells. Total number of ablations; n = 71 (16 h), n = 55 (24 h), n = 22 (32 h), n = 22 (38 h). (D-I) Rarely induced restorative divisions in outer adjacent cells did not trigger cell fate change in any of the daughter cells. After 16 h and 38 h of cortex ablation, expression pattern of *WER::GFP* in outer adjacent periclinally divided daughter cells did not change (D and E). Expression patterns of LRC marker *E4722* (F, 16 h; n = 52/52, 38 h; n = 43/45), *SCR::SCR-YFP* (G, 16 h; n = 24/24, 38 h; n = 37/37), and *WER::GFP* (H and I) after 16 h and 38 h of ablation did not change in the presence of 1 μM NAA, which increased occurrence of restorative divisions at the outer adjacent side. Relative fluorescence signal was calculated as follows; *WER::GFP* signals in the periclinally divided cells were divided by non-periclinally divided epidermal cells. Sample numbers are as follows; in D and E (16 h; n = 14, 38h; n = 9), in H and I (16 h; n = 10, 38h; n = 7). The error bars show SD. Red asterisks: sites of ablation. Roots were stained with propidium iodide.

After ablation of cortex cells, we monitored the endodermis-specific marker *SCR::SCR-YFP* and the cortex-specific marker *Co2::HYFP* (Heidstra et al., 2004) to analyze the cell-fate changes after restorative divisions. Also, in this case, the outer daughter cell gradually lost its endodermal fate, as evidenced by decreasing SCARECROW (SCR)-YFP expression (Figures 4I and S4A), and acquired the cortex fate (increasing *Co2::HYFP* expression) at about 16–24 h after ablation (Figures 4E and 4I).

We also followed cell-fate changes after elimination of an endodermal cell, which is replaced by the restorative division of the adjacent pericycle cell using endodermal *SCR::SCR-YFP* and *SHR::SHR-GFP* that is more uniformly distributed in stele, including pericycle cells, but is restricted to the nucleus specifically in endodermis (Nakajima et al., 2001). Analogically to other cell types, we observed that the newly formed outer daughter cell gradually changed its fate to become endodermis as manifested by the activation of SCR-YFP expression (Figures 4F, 4J, and S4B) and nuclear appearance of SHORTROOT (SHR)-GFP (Figure S4C).

In rare cases (well below 10% of events), cells at the outer side of the wound would divide periclinally in addition to cells at the inner side. Notably, in such cases, we did not detect any changes in cell fate of the daughter cells using *SCR::SCR-YFP*, *WER::GFP* and *E4722* marker lines (Figures S4D–S4I), contrasting with consistent cell fate re-specifications at the inner-wound side. This suggests that an inside-to-outside intercellular signaling is required for correct re-specification during wound regeneration.

Taken together, restorative periclinal divisions induced by wounding generate daughter cells that replace dead or damaged cells by acquiring a new, “correct” cell fate. Thus, restorative cell division represents a special case of formative division. This necessitates the existence of multiple intercellular signaling mechanisms, constantly providing positional information to the cells, which acts also outside the stem cell niche.

### Re-activation of SHR/SCR-CYCD6 Stem Cell Program Replaces Eliminated Cortex

**Figure 5:**
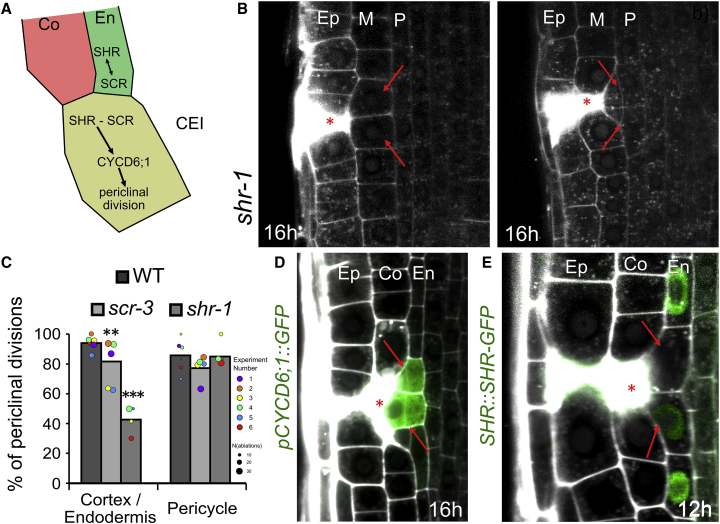
Re-activation of SHR/SCR-CYCD6;1 Module during Restoration of Cortex (A) SHR/SCR-CYCD6;1 module in ground tissue development (Fisher and Sozzani, 2016, Sozzani et al., 2010). (B and C) Ablation of epidermal cells (B, left) and ground tissue (B, right) in *shr-1* mutant. Quantification of periclinal divisions in *scr-3* and *shr-1* (C). Data are represented as weighted mean (bar) and individual experiments (dots, area indicates sample size); p values from CLR for *scr-3*: 0.00234; *shr-1*: 8.61E−9. M, ground tissue layer formed in *shr* mutants. (D and E) Expression of *CYCD6;1::GFP* is upregulated in periclinally divided endodermis cell after ablation of cortex (for the translational fusion, see Figure S5A) (D), while *SHR::SHR-GFP* is downregulated before periclinal division (see also Figure S5B) (E). Total number of ablations: n = 109 (CYCD6;1), n = 101 (SHR). Red asterisks: sites of ablation. Roots were stained with PI. See also Figure S5 and Table S1.

How do the cells adjacent to the wound get activated and generate cells with new, correct identities during wound healing? The asymmetric cell division generating different cell types occurs typically only in the stem cell niche. For example, cortex and endodermis cell files originate from the same stem cell, the cortex-endodermis initial (CEI). The CEI continuously divides anticlinally to renew itself and produce a daughter cell, which further divides periclinally to generate cortex and endodermis (De Rybel et al., 2016, Helariutta et al., 2000, Kumpf and Nowack, 2015, Scheres et al., 2002). This process is governed by the transcription factors SHR and SCR, which activate the expression of CYCD6;1 and thus initiate a switch in division plane orientation (Heidstra et al., 2004, Sozzani et al., 2010; Figure 5A). Hence, *scr* and *shr* mutants lacking the initial formative division contain only a single layer of ground tissue, while overexpression of SHR or CYCD6;1 leads to the formation of additional ground-tissue cell layers (Benfey et al., 1993, Sevilem et al., 2015). Despite SHR and SCR being expressed throughout the whole-root meristem, activation of CYCD6;1 and formative cell division within young seedlings occurs only in the stem cell niche (Cruz-Ramírez et al., 2012, Heidstra et al., 2004, Sozzani et al., 2010, Yu et al., 2017).

**Figure S5:**
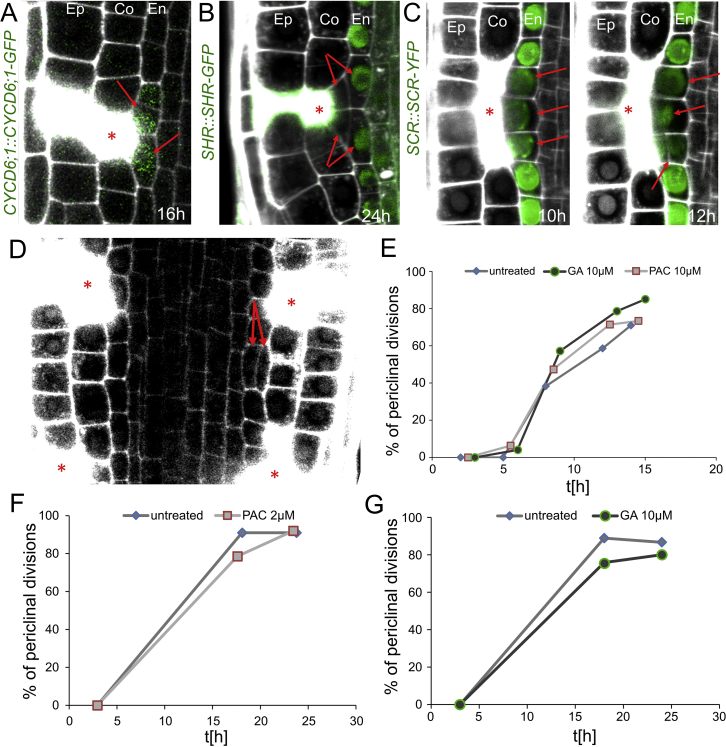
Periclinal Divisions in Endodermis Are Independent of GA-Inducible Activation of Middle-Cortex Formation, Related to Figure 5 (A) Ablation of cortex is followed by upregulation of *CYCD6;1* translational fusion before induction of periclinal division in the inner adjacent endodermis cells. (B and C) After cortex ablation, downregulation of *SHR::SHR-GFP* is followed by induction of periclinal division in endodermis cells outside of the stem cell niche (B), which is similar to downregulation of *SCR::SCR-YFP* in endodermal cells undergoing periclinal divisions (C). (D) Treatment of 10 μM GA resulted in ectopic inductions of periclinal divisions which are independent from cortex ablations as seen in root meristem 1 h after ablation. Note that the periclinal division is not adjacent to ablations (red arrows). (E-G) Effect of GA or PAC on the rate of periclinal divisions in endodermis. For the short-term observation, 10 μM GA and PAC (E), for the long-term observation, 2 μM PAC (F) and 10 μM GA (G) were used. There is no influence of the treatments on the division rates in endodermis cells. Total number of ablations; n = 30-40 per treatment. Red asterisks: sites of ablation. Roots were stained with propidium iodide.

We have examined involvement of the SHR/SCR-CYCD6;1 module in the restorative division. In the *shr* and *scr* mutants, the restorative cell divisions specifically in the single ground tissue layer were significantly defective, whereas the division of other cell types occurred normally (Figures 5B and 5C). Notably, *CYCD6;1::GFP* and *CYCD6;1::CYCD6;1-GFP* expression, typically detectable only in the stem cells, was upregulated specifically in endodermal cells next to eliminated cortex cells and started between 3 and 7 h after wounding well before the onset of the restorative division (Figures 5D and S5A; Video S4). On the other hand, SHR-GFP and SCR-YFP signal intensity decreased before and during mitosis (Figures 5E, S5B, and S5C). A similar downregulation of SCR/SHR and activation of CYCD6;1 expression can be observed outside of the stem cell niche during initiation of an additional cortex layer, so called middle cortex (Benfey et al., 1993, Paquette and Benfey, 2005). Middle-cortex formation shares some similarities with restorative divisions of endodermis, such as switch of cell division plane to periclinal. However, it occurs only in older roots and is under a strong control of the phytohormone gibberellic acid (GA) (Cui and Benfey, 2009, Gong et al., 2016, Paquette and Benfey, 2005). Notably, neither application of GA nor GA biosynthesis inhibitor paclobutrazol (PAC) had an influence on the competence of endodermis cells to undergo restorative divisions (Figures S5D–S5G), implying that restorative division and middle-cortex formation while both depending on SHR/SCR-CYCD6;1 signaling use at least partly divergent mechanisms.

Video S4. Ablation of Cortex Cells Leads to Specific Induction of CYCD6;1 in Adjacent Endodermis Cells before and during Restorative Divisions, Related to Figure 5

Our observations show that cortex elimination by wounding activates the endodermis/cortex SHR/SCR-CYCD6;1 stem cell program in the underlying endodermis cell, enabling its restorative division and ultimately healing the wound by generating daughter cells with the correct fate.

### Re-activation of FEZ/SMB Stem Cell Program Replaces Eliminated Epidermis

Besides CEIs, the stem cell niche consists of other initials that divide asymmetrically to form distinct cell types, such as the LRC/epidermis initials (LEIs).

**Figure 6:**
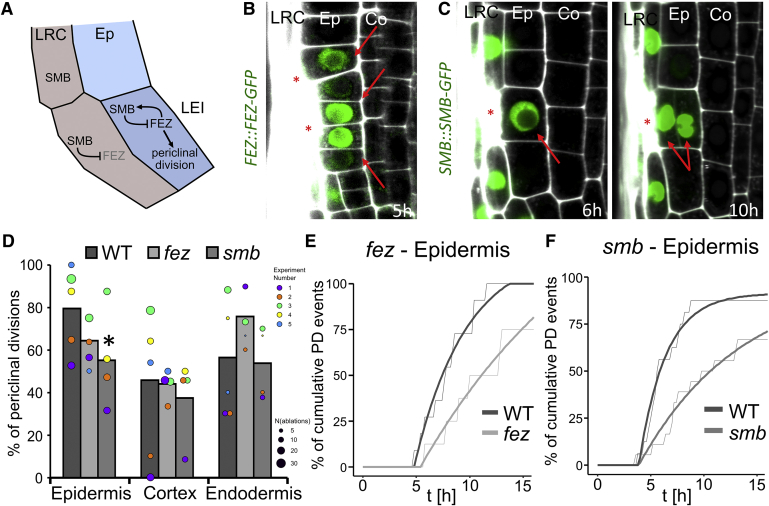
Re-activation of FEZ/SMB Module during Restoration of LRC (A) FEZ/SMB module in LRC development (Fisher and Sozzani, 2016, Willemsen et al., 2008). Gray font shows reduced expression. (B and C) Expression of *FEZ::FEZ-GFP* (B) and *SMB::SMB-GFP* (C) is upregulated in epidermal cells after ablation of LRC (see also Figures S6A and S6B). (D) Quantification of periclinal divisions after ablation in *fez* and *smb* mutants in different layers show reduced numbers in mutant epidermis cells. Data are represented as weighted mean (bar) and individual experiments (dots, area indicates sample size); p values from CLR: *fez*: 0.075 (epidermis), 0.50 (cortex), 0.077 (endodermis), *smb*: 0.013 (epidermis), 0.79 (cortex), 0.73 (endodermis). (E and F) Cumulative divisions over time in *fez* (E) and *smb* (F) monitored by vertical stage microscopy. Observed ablations; n = 20 per genotype. Thin lines show raw data and thick lines show fitted curves calculated by least-square estimates (y = a − b^∗^eˆ(c^∗^x)). See also Figure S6C. Red asterisks: sites of ablation. Roots were stained with PI. See also Figure S6.

Their correct division pattern is regulated by two transcription factors, FEZ and SMB (Fisher and Sozzani, 2016, Willemsen et al., 2008). FEZ induces the asymmetric division of the initial, while SMB inhibits further divisions of already specified LRC cells. *fez* mutants lack additional stem cell divisions and show decreased number of LRC layers, while stem cells in *smb* mutant undergo more periclinal divisions and generate additional LRC layers (Fisher and Sozzani, 2016, Willemsen et al., 2008). The concomitant expression of both proteins is specific to LRC/epidermis initials in the stem cell niche and very young LRC cells, where they interact with each other in a negative feedback loop, and their expression is never observed in already specified epidermis cells (Figure 6A).

**Figure S6:**
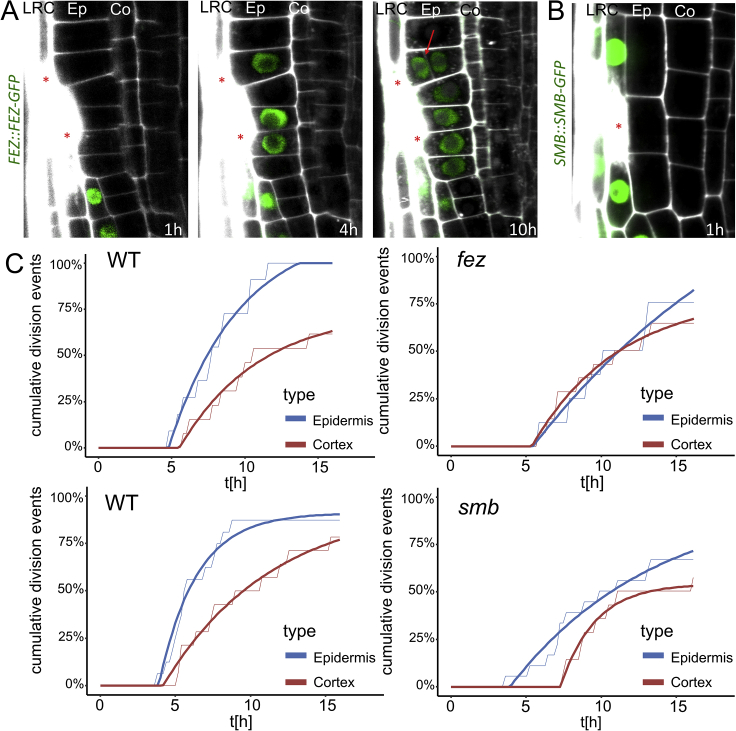
Periclinal Divisions in Epidermis Require Functional FEZ/SMB Module, Related to Figure 6 (A and B) Additional time points for the ablations shown in Figures 5B and 5C. While expression of *FEZ::FEZ-GFP* (A) and *SMB::SMB-GFP* (B) is absent from epidermis cells outside of the stem cell niche, they are upregulated in the adjacent epidermis cells after ablation of LRC cells, which gradually triggers periclinal divisions. (C) Rate of periclinal division in cortex cells remains unchanged in mutants, while rates of periclinal divisions in epidermis decreased as can be seen in cumulative division events over time. Observed roots; n = 10 with 2 ablation sites per genotype; exponential curves: y = a-b^∗^eˆ(c^∗^x). Thin lines show raw data and thick lines show fitted curves calculated by least square estimates. Red asterisks: sites of ablation. Roots were stained with propidium iodide.

We observed a specific upregulation of both *FEZ::FEZ-GFP* and *SMB::SMB-GFP* in epidermis cells adjacent to wounded LRC cells (Figures 6B, 6C, S6A, and S6B). The upregulation started 4–6 h after LRC elimination and was always followed by a periclinal division. While the FEZ expression dropped shortly after division, SMB was asymmetrically upregulated in the newly forming LRC cells, and it vanished in the remaining epidermis cells (see Figure 4B). In the *fez* mutant, we observed a reduction in epidermis division rate and an increase in the rates of cortex and endodermis (Figures 6D and 6E). In the *smb* mutant, the rate of epidermis division following LRC elimination dropped significantly, whereas other cell types showed normal rate of restorative divisions (Figures 6D and 6F).

These observations revealed a specific upregulation and functional requirement of both these stem cell regulators for the restorative cell division of epidermis, leading to the replacement of the eliminated LRC by daughter cells with a correct cell fate. Thus, as in case of cortex replacement, the re-activation of the specific stem cell program mediates also the replacement of LRC during wound healing.

## Discussion

Plants evolved distinct mechanisms of patterning compared to animals due to the constraints of immobile cells encapsulated within cell walls. Owing to their sessile lifestyle, they also frequently must endure injuries, but little is known of how the tissue is restored after wounding.

In this work, we address the mechanisms underlying wound healing and pattern restoration using targeted laser elimination of different cell types coupled to prolonged live-cell imaging. This approach allowed us to identify in multiple plant species the process of restorative patterning, which encompasses restorative cell divisions initiated after injury, and subsequent *de novo* specification of the correct cell fates and ultimately leads to replacement of the eliminated cells and correct regeneration of the injured tissues.

### Restorative Cell Divisions Specifically and Correctly Replace Eliminated Cells

We have shown here that root cells disrupted by injury are not simply replaced by a proliferation of healthy cells from the same cell file adjacent above and below to the wound. Instead, inner adjacent cells become activated to replace the dead neighbor by a process we call restorative patterning. This special type of formative cell division involves several coordinated processes: (1) recognition of the disrupted tissue by inner adjacent cells, (2) accelerated entry into and progress through the cell cycle, (3) re-orientation of the cell division plane, and (4) correct cell-fate re-specification of the generated daughter cell, which fills the wound. Furthermore, restorative divisions in inner adjacent cells were observed independently of the nature of tissue disruption: ablation of single or multiple cell layers, drug-induced cell death, and natural collapse of multiple cells.

**Figure S7:**
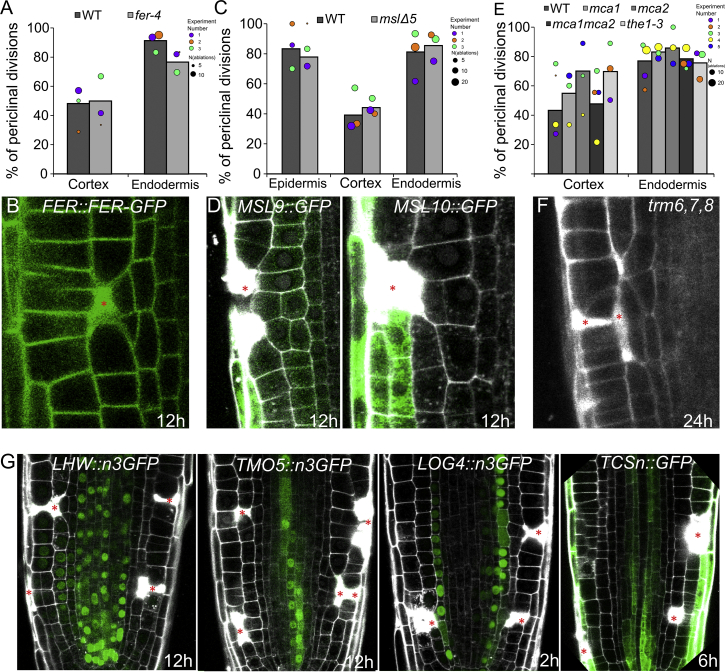
Role of Known Components for Mechanosensing, Induction of Periclinal Division, and Preprophase Band during Restorative Divisions, Related to Figure 1 (A-E) Known mechanosensors FERONIA (FER), MECHANOSENSITIVE SMALL CONDUCTANCE-LIKE channels (MSLs), MID1-COMPLEMENTING ACTIVITY channels (MCAs) and THESEUS1 (THE1) are not involved in restorative cell divisions. (A) Quantification of periclinal divisions in *fer-4* mutants 12 h after ablation. Data are represented as weighted mean (bar) and individual experiments (dots, area indicates sample size). (B) Expression pattern of *FER::FER-GFP* during restorative division is unchanged. Autofluorescence from PI staining marks dead cells in the GFP channel. (C) Quantification of periclinal divisions 12 h after ablation in *mslΔ5* quintuple mutants. Data are represented as weighted mean (bar) and individual experiments (dots, area indicates sample size). (D) Expression of *MSL9::MSL9-GFP* (left) and *MSL10::MSL10-GFP* (right) in the *mslΔ5* background did not change during restorative divisions. (E) Quantification of periclinal divisions 12 h after ablation in *mca1, mca2, mca1mca2, the1-3* mutants. Data are represented as weighted mean (bar) and individual experiments (dots, area indicates sample size). (F) Preprophase band (PPB) does not define the cortical division site (CDS) in restorative divisions. *trm6trm7trm8* triple mutant, which lacks the PPB, does not display defects in deposition of periclinal cell walls in long term imaging (24 h) during vertical stage experiments. (G) Positive regulators of periclinal cell division LONESOME HIGHWAY (LHW), TARGET OF MONOPTEROS5 (TMO5), their downstream targets LONELY GUY4 (LOG4) and cytokinin-responsive TCS are not involved in restorative divisions in multiple cell types as can be seen in: *pLHW::n3GFP*, *pTMO5::n3GFP*, *pLOG4::n3GFP* and *pTCSn::GFP* (from left to right) at indicated time points. Red asterisks: sites of ablation. Roots were stained with propidium iodide.

The underlying signaling cascades for restorative patterning remain largely elusive; however, we provide some initial insights. The known components of mechanosensing, periclinal cell divisions, and preprophase band formations do not seem to be involved (Figure S7). On the other hand, the graded expression of PLT transcription regulators seems to provide some cell types of the root with their regenerative competence.

Our observations also show that the main fraction of dividing cells finish mitosis between 5 and 8 h, the minimum time frame required for cell divisions in plants and other eukaryotes (Mickelson-Young et al., 2016). Therefore, signaling from the wound to initiate cell-cycle transitions in inner adjacent cells must happen very rapidly after tissue disruption and likely relies on non-genomic factors, such as mechanical properties and local geometry of disrupted tissues.

The switch in the division plane is a crucial part of the restorative patterning. Notably, it has been shown that mechanical perturbations, including cell ablation, in the shoot apical meristem can trigger division plane re-orientation of surrounding cells (Louveaux et al., 2016), indicating that the process of restorative patterning and the coordinated cell division plane switching, here characterized in the root, may operate more globally throughout the plant.

### Re-activation of Stem Cell Mechanisms Mediates Restorative Patterning

A remarkable feature of restorative patterning is that cells undergoing proliferative divisions with already clearly specified fates start to perform formative divisions and undergo cell-fate re-specification. This allows the generation of daughter cells with correct cell fates to regenerate the disrupted tissue.

In the cell-type lineages, which share common ancestors in the stem cell niche, such as LEIs and CEIs, we observed an activation of regulators usually specific only to the respective stem cells. The upstream activators of these stem cell pathways await identification, but the genetic analysis revealed that restorative patterning is much less efficient albeit not completely prevented in mutants defective in those stem cell regulators. Nonetheless, restorative divisions where the ablated and the activated cell do not have a common ancestor (especially cortex division after epidermis ablation) still occur but with reduced efficiency in division plane switching and wound healing. This implies some redundancy in mechanisms for restorative patterning and indicates the existence of a slower mechanism, which potentially operates in all cell types regardless of their stem cell ancestry. Therefore, the additional activation of the stem cell regulators, which were shown to ectopically induce periclinal divisions by overexpression (Sozzani et al., 2010, Willemsen et al., 2008, Yu et al., 2017), provides increased efficiency and robustness to this so-far unidentified, “default” cell-type-independent restorative mechanism.

For the part of restorative patterning mechanism that involves stem cell pathway activation, one can envision that even already specified cells preserve a “memory” of their stem cell ancestry and, upon receiving a wound, signal re-activate the stem cell pathway. However, the basal, default restorative patterning implies the existence of global positional signaling mechanism(s) that determine which cell fate the daughter cells should adopt following restorative division. While the cell fates of inner, wound-adjacent cells of ablation is re-specified, the cell fate of occasionally dividing outer adjacent cells is not affected, suggesting that the positional signaling mediating the coordinated cell-fate changes occurs in a radial direction from inside to outside. Thus, the mechanism of wound healing by restorative divisions starts always from the inner tissues regardless of the wounding process.

As suggested previously (van den Berg et al., 1995, Berger et al., 1998, Kidner et al., 2000), such positional signaling would operate outside the stem cell niche and thus constantly allow already-specified cells to adapt their cell fate according to their position within the tissue architecture. In the case of endodermis specification, this is likely the well-known SHR/SCR radial signaling module that is expressed and operates throughout the whole-root meristematic zone (Heidstra et al., 2004), but for other cell types, any notion of this signaling remains elusive.

### Conclusion

In summary, this work provides insights into the plant-specific mechanism underlying wound healing. Focusing on the coordinated response of already specified root cells to the local injury, we uncovered a process of restorative patterning, which correctly replaces eliminated cells and allows plant tissues to heal despite the absence of cell migration, which is the basis of wound healing in animals. The nature of the wound signal remains enigmatic, but the downstream restorative patterning involves re-activation of stem cell-specific signaling pathways inducing asymmetric, formative divisions and cell-fate re-specifications ultimately closing and healing the wound. In addition to the regenerative competence requiring and correlating with the gradually decreasing expression of PLT transcription factors, our result also suggested the existence of undiscovered signaling mechanisms constantly conveying positional information throughout all tissues of the root meristem.

Besides these insights into the mechanism of wound healing, our method of laser-assisted local tissue perturbation coupled with long-term, high-resolution imaging during vertical growth opens new possibilities to address the mechanism of intercellular signaling and patterning in plant tissues. Thus, further studies into restorative patterning using molecular genetics, single-cell transcriptomics, and mechanical modeling, among other approaches will reveal not only a broader understanding of the mechanism of wound healing, but also how plants establish and maintain their body patterns.

## STAR★Methods

### Key Resources Table

**Table:** 

REAGENT or RESOURCE	SOURCE	IDENTIFIER
**Chemicals, Peptides, and Recombinant Proteins**		

Propidium iodide	Sigma-Aldrich	Cat#P4864
Propidium iodide	Thermo Scientific	Cat#P3566
Hydroxyurea	Sigma-Aldrich	Cat#H8627
Gibberellic acid	Sigma-Aldrich	Cat#G7645
Paclobutrazol	Sigma-Aldrich	Cat#46046
Epibrassinolide	Sigma-Aldrich	Cat#E1641
Dexamethasone	Sigma-Aldrich	Cat#D1756
1-Naphthylacetic acid	Sigma-Aldrich	Cat#N0640
6-Benzylaminopurine	Sigma-Aldrich	Cat#B3408

**Experimental Models: Organisms/Strains**		

*Arabidopsis:* WT Col-0	https://www.ncbi.nlm.nih.gov/Taxonomy/Browser/wwwtax.cgi	NCBI:txid3702
*Capsella rubella*	https://www.ncbi.nlm.nih.gov/Taxonomy/Browser/wwwtax.cgi	NCBI:txid81985
*Nicotiana benthamiana*	https://www.ncbi.nlm.nih.gov/Taxonomy/Browser/wwwtax.cgi	NCBI:txid4100
*Oryza sativa*	https://www.ncbi.nlm.nih.gov/Taxonomy/Browser/wwwtax.cgi?mode=Info&id=4530&lvl=3&lin=f&keep=1&srchmode=1&unlock	NCBI:txid4530
*Arabidopsis: cre1-12 ahk2-2 ahk3-3*	Higuchi et al., 2004	Cross between *cre1-12* (SALK_048970), *ahk2-2* and *ahk3-3* (SALK_069269)
*Arabidopsis: plt1-4, plt2-2*	Aida et al., 2004	N/A
*Arabidopsis: plt1plt2*	Blilou et al., 2005	Cross between *plt1-4* and *plt2-2*
*Arabidopsis: wind1wind2wind3wind4*	Iwase et al., 2011	Cross between wind1 (SALK_027272), wind2 (SALK_139727), wind3 (SALK_091212) and wind4 (SALK_099481)
*Arabidopsis: shr-1*	Benfey et al., 1993	NASC ID: N3997
*Arabidopsis: scr-3*	Fukaki et al., 1996	NASC ID: N3997
*Arabidopsis: fez-1*, *smb-1*	Willemsen et al., 2008	N/A
*Arabidopsis: fer-4*	Duan et al., 2010	GK-106A06
*Arabidopsis: msl4msl5msl6msl9msl10* (*mslΔ5*)	Haswell et al., 2008	Cross between *msl4-1* (SALK_142497), *msl5-2* (SALK_127784), *msls6-1* (SALK_06711), *msl9-1* (SALK_114626), *msl10-1* (SALK_076254)
*Arabidopsis: mca1, mca2*	Yamanaka et al., 2010	*mca1* (N/A) *mca2* (SALK_129208)
*Arabidopsis: mca1mca2*	Yamanaka et al., 2010	Cross between *mca1* and *mca2*
*Arabidopsis: the1-3*	Hématy et al., 2007	FLAG_201_C06
*Arabidopsis: trm6trm7trm8*	Schaefer et al., 2017	Cross between *trm6-1* (GK_048G03), *trm7-1* (SALK_074058), *trm8-1* (SALK_150274)
*Arabidopsis: 35S::PLT2-GR, PLT1::ECFP, PLT2::YFP*	Galinha et al., 2007	Transgenic Col-0
*Arabidopsis: HTR2::CDT1-GFP*	Yin et al., 2014	Transgenic Col-0
*Arabidopsis: CycB1;1::GFP*	Ubeda-Tomás et al., 2009	Transgenic Col-0
*Arabidopsis: KN::GFP-KN*	Reichardt et al., 2007	Transgenic Ler/Nd
*Arabidopsis: SCR::SCR-YFP, Co2::HYFP*	Heidstra et al., 2004	Transgenic N/A
*Arabidopsis: SHR::SHR-GFP*	Nakajima et al., 2001	Transgenic Col-0
*Arabidopsis: J0571*	Haseloff, 1999	NASC ID: N9094
*Arabidopsis: WER::GFP*	Lee and Schiefelbein, 1999	NASC ID: N66493
*Arabidopsis: RBR1::RBR1-GFP*	Magyar et al., 2012	Transgenic Col-0
*Arabidopsis: ERF115::GFP-NLS, PAT1::GFP-NLS*	Heyman et al., 2016	Transgenic Col-0
*Arabidopsis: ERF115-SRDX*	Heyman et al., 2013	Transgenic Col-0
*Arabidopsis: CYCD2;1::CYCD2;1:GFP*	Sanz et al., 2011	Transgenic Col-0
*Arabidopsis: WIND1::GFP, 35S::WIND1-SRDX, 35S::WIND1*	Iwase et al., 2011	Transgenic Col-0
*Arabidopsis: SMB::SMB-GFP*, *FEZ::FEZ-GFP*	Willemsen et al., 2008	Transgenic Col-0
*Arabidopsis: CYCD6;1::GFP*	Sozzani et al., 2010	Transgenic Col-0
*Arabidopsis: E4722*	Gifford et al., 2008	NASC ID: N70265
*Arabidopsis:MSL9::GFP-GUS*/*mslΔ5, MSL10::GFP-GUS*/*mslΔ5*	Haswell et al., 2008	Transgenic Col-0
*Arabidopsis: FER::FER-GFP/fer-4*	Li et al., 2015	Transgenic Col-0
*Arabidopsis: TMO5::n3GFP, LHW::n3GFP*	De Rybel et al., 2013	Transgenic Col-0
*Arabidopsis: LOG4::n3GFP*	De Rybel et al., 2014	Transgenic Col-0
*Arabidopsis: TCSn::GFP*	Zürcher et al., 2013	NASC ID: N69180

**Software and Algorithms**		

imageJ	https://imagej.net/Welcome	RRID:SCR_003070
Zeiss Zen 2011	https://www.zeiss.com/	N/A
R project	http://www.r-project.org/	RRID:SCR_001905
R-studio	https://www.rstudio.com/	RRID:SCR_000432
ggplot2	https://ggplot2.tidyverse.org/	RRID:SCR_014601
survival	https://cran.r-project.org/web/packages/survival/survival.pdf	N/A
minipack.lm	https://www.rdocumentation.org/packages/minpack.lm/versions/1.2-1/topics/nlsLM	N/A
TipTracker	https://elifesciences.org/articles/26792/figures#SD2-data	N/A

### Contact for Reagent and Resource Sharing

Further information and requests for resources and reagents should be directed to and will be fulfilled by the Lead Contact, Jiří Friml (jiri.friml@ist.ac.at).

### Experimental Model and Subject Details

#### Plant material

*Arabidopsis thaliana* (L.) Heynh (accession Columbia-0), *Nicotiana benthamiana, Capsella rubella* and *Oryza sativa* were used in this work. The transgenic *Arabidopsis thaliana* lines and mutant lines were described previously: *cre1-12 ahk2-2 ahk3-3* (Higuchi et al., 2004), *plt1-4* and *plt2-2* (Aida et al., 2004), *plt1plt2* (Blilou et al., 2005), *35S::PLT2-GR, PLT1::ECFP, PLT2::YFP* (Galinha et al., 2007), *HTR2::CDT1a-GFP* (Yin et al., 2014), *pCYCB1;1::GFP* line (Ubeda-Tomás et al., 2009), *KN::GFP-KN* (Reichardt et al., 2007), *SCR::SCR-YFP* and *Co2::HYFP* (Heidstra et al., 2004), *SHR::SHR-GFP* (Nakajima et al., 2001), *J0571* (Haseloff, 1999), *WER::GFP* (Lee and Schiefelbein, 1999), *shr-1* (Benfey et al., 1993), *scr-3* (Fukaki et al., 1996), *RBR1::RBR1-GFP* and *E2FA::E2FA-GFP* (Magyar et al., 2012), *ERF115::GFP-NLS, PAT1::GFP-NLS* (Heyman et al., 2016), *ERF115-SRDX* (Heyman et al., 2013), *CYCD2;1::CYCD2;1:GFP* (Sanz et al., 2011), *WIND1::GFP, wind1wind2wind3wind4, 35S::WIND1-SRDX, 35S::WIND1* (Iwase et al., 2011) *SMB::SMB-GFP*, *FEZ::FEZ-GFP*, *fez-1* and *smb-1* (Willemsen et al., 2008), *CYCD6;1::GFP* (Sozzani et al., 2010), E4722 (Gifford et al., 2008), *fer-4* (Duan et al., 2010), *msl4msl5msl6msl9msl10* (*mslΔ5*), *MSL9::GFP-GUS*/*mslΔ5, MSL10::GFP-GUS*/*mslΔ5* (Haswell et al., 2008), *mca1, mca2, mca1mca2* (Yamanaka et al., 2010), *the1-3* (Hématy et al., 2007), *FER::FER-GFP* (Li et al., 2015), *trm6trm7trm8* (Schaefer et al., 2017), *TMO5::n3GFP, LHW::n3GFP* (De Rybel et al., 2013), *LOG4::n3GFP* (De Rybel et al., 2014) *TCSn::GFP* (Zürcher et al., 2013).

#### Growth conditions

Seeds of *A. thaliana* were sown on Murashige and Skoog (1/2MS) medium (Duchefa) with 1% sucrose and 0.8% - 1% agar, stratified for 2 d and grown for 4-7 d at 21°C in a 16 h light/8 h dark cycle.

### Method Details

#### Pharmacological treatments

Seedlings were transferred on solid MS medium with the indicated chemicals: propidium iodide (PI, 10 μM, Sigma-Aldrich or Thermofisher), hydroxyurea (HU, final concentration 5 mM, Sigma-Aldrich) for 24 hours, gibberellic acid (GA, final concentration 10 μM, Sigma-Aldrich) for 1 hour before ablation, paclobutrazol (PAC, final concentration 2 or 10 μM as indicated, Sigma-Aldrich) for 1 hour before ablation, epibrassinolide (EBL, Sigma Aldrich, final concentration 1 μM) for 1 hour before ablation, dexamethasone (DEX, Sigma Aldrich, final concentration 5 μM) for 1 hour before ablation, 1-Naphthylacetic acid f (NAA, Sigma Aldrich, final concentration 1 μM) or 1 hour before ablation, 6-Benzylaminopurine (BAP, Sigma Aldrich, final concentration 50 nM) for 1 hour before ablation.

#### Sample preparation

Seedlings were placed on chambered cover glass (VWR, Kammerdeckgläser, Lab-Tek, Nunc - eine kammer, catalog number: 734-2056) as described (Marhavý and Benková, 2015). Using the chamber, we cut out the block of solid MS media, added propidium iodide on it, let it soak, transferred 10-15 seedlings on it and put them together to a chamber.

#### Confocal imaging and image processing

Confocal imaging was performed with Zeiss LSM700/800 inverted microscopes or Leica SP5 upright microscope. Pictures were taken by 20x or 40x objectives. Fluorescence signals for GFP (excitation 488 nm, emission 507 nm), YFP (excitation 514 nm, emission 527 nm) and PI (excitation 536 nm, emission 617 nm) were detected. Samples were observed after 16 hours of ablation or at indicated time points. Images were analyzed using the ImageJ (NIH; https://imagej.nih.gov/ij) and Zeiss Zen 2011 software. Where necessary, images were processed using the ‘sharpen’ tool to produce clearer images of cellular organization.

#### Vertical stage microscopy and root tracking

Vertical stage microscopy for long-term tracking (usually 24 hours) of root meristems was performed as described (von Wangenheim et al., 2017). Roots were imaged with a vertically positioned LSM700 inverted confocal microscope and Zeiss Zen 2011 software with 20x objective and detection of PI, GFP (see above) and transmitted light. For observation of the whole root meristem, z stacks of 42 μm were set accordingly. For the root-tracking, the TipTracker MATLAB script was used with default settings except for interval duration 720 s (12 min) and number of time points 120. The resulting images were concatenated and analyzed using ImageJ.

#### UV laser ablation setup

The UV laser ablation setup is based on the layout published in ref. (Colombelli et al., 2004) and also described in ref. (Marhavý et al., 2016) that uses a passively Q-switched solid-state 355-nm UV-A laser (Powerchip, Teem Photonics) with a pulse energy of 15 μJ at a repetition rate of 1 kHZ. With a pulse length of < 350 psec, a peak power of 40 kW was obtained, of which typically < 5% was used to cut tissue. The power was modulated with an acousto-optic modulator (AOM; Pegasus Optik, AA.MQl l0-43-UV). The laser beam diameter matched the size of the back aperture of the objectives by means of a variable zoom beam expander (Sill Optics), enabling diffraction-limited focusing while maintaining high transmission for objectives with magnifications in the 20 × to 100 × range. Point scanning was realized with a pair of high-speed galvanometric mirrors (Cambridge Technology, Lightning DS). To this end, the scanning mirrors were imaged into the image plane of the rear port of a conventional inverted microscope (Zeiss, Axio Observer Z1) with a telecentric f-θ objective (Jenoptik). To facilitate adjusting parfocality between the cutter and the spinning disk and compensate for the offset between the positions of the back planes of different objectives, the scan mirrors and the scan optics were mounted on a common translation stage. In the microscope reflector cube, a dichroic mirror reflected the UV light onto the sample but transmitted the fluorescence excitation and emission light. A UV-blocking filter in the emission path protected the camera and enabled simultaneous imaging and ablation. The AOM, the galvanometric mirrors, and a motorized stage (ASI, MS 2000) with a piezo-electric actuator on which the sample was mounted were computer controlled by custom-made software (Labview, National Instruments), enabling three-dimensional cuttings. The maximum field size for diffraction-limited cutting with little geometric distortion, high homogeneity of the intensity, and good field flatness was 300 × 300 m^2^ for a 40 × objective. The maximum depth was limited by the free-working distance of the objective used and the travel of the piezo-actuator (100 μm).

### Quantification and Statistical Analysis

For counting the PD events, at each ablation site, the occurrence of a periclinal division (marked by a division plane parallel or oblique to the growth axis) in any of the adjacent inner cells was recorded as 1/1 or 0/1 in case of only anticlinal divisions occurring.

The binary outcome “Periclinal division happened yes/no” of the ablations experiments was by definition distributed in a binomial manner, hence statistical tests for binary values are required. Based on the observation that periclinal division rates varied highly between different experiments but trends of lower division rates between mutants/treatments and controls were always visible within single experiments, we decided to opt for a statistical test that accounts for paired datasets (paired within individual experiments). Hence, the statistical significance was evaluated with conditional logistic regression (CLR) after ref. (Kleinbaum and Klein, 2010). and ref. (Campbell, 2006). “Periclinal division happened” was used as binary input for each observed ablation site in the clogit function from the R package “survival.” Data was paired with experiment number as stratum. We assume that external factors such as daytime, growth medium batch and propidium iodide batch are major causes for variations between experiments.

In the case of vertical stage microscopy with root tracking, division events were counted by marking the time point at which a new cell wall (stained by propidium iodide) appeared in the first, inner adjacent cell of the ablation site. For ablations, only periclinal divisions (vertical cell walls) were counted whereas for the virtual ablations both anticlinal and periclinal divisions were quantified. The virtual ablation sites were arbitrarily chosen similar as in the laser ablation experiments and the division events were counted on videos after registration (correct 3D drift tool in ImageJ). The percentage of cumulative division events over time was plotted using the R studio. Non-linear regression curves for each experiment were calculated using the nlsLM function of the R package “minipack.lm” assuming an asymmetric sigmoidal (y = a ^∗^eˆ(-b^∗^eˆ(-c^∗^x))) or exponential (y = a-b^∗^eˆ(c^∗^x)) behavior with arbitrarily chosen starting parameters. The calculated final parameters with lowest residual sum-of-squares were chosen to determine the first derivation of the fitted curves to estimate the probability of division events over time.

Asterisks illustrate the p value: p < 0.001 is ^∗∗∗^, p < 0.01 is ^∗∗^ and p < 0.05 is ^∗^

Number of repetitions and replicates are mentioned for each experiment in the legends.

## Supplemental Information

Table S1. Total Number of Generated Ablations Supporting Individual Statements, Related to Figures 1, 2, 3, 4, 5, and 6
