# Supplementary material for: Re-activation of Stem Cell Pathways for Pattern Restoration in Plant Wound Healing
Source: Cell. 2019 May 2;177(4):957–969.e13. doi: 10.1016/j.cell.2019.04.015 (PMC6506278; doi:10.1016/j.cell.2019.04.015)
Supplement: Table S1. Total Number of Generated Ablations Supporting Individual Statements, Related to Figures 1, 2, 3, 4, 5, and 6 [file mmc1.pdf]

Cell, Volume *177*

Supplemental Information

Re-activation of Stem Cell Pathways

for Pattern Restoration in Plant Wound Healing

Petra Marhava, Lukas Hoermayer, Saiko Yoshida, Peter Marhavý, Eva Benková, and Jiří Friml

SUPPLEMENTAL INFORMATION

Supplemental Tables

Table S1. Total number of generated ablations supporting individual statements, Related to Figures 1-6 and S1-7.

**Table S1.**		
Observation	Reference	Total number of ablations
Cell types	Fig 1D-G	125
Competence	Fig 2A-B	505
35S::PLT-GR	Fig 2C-D	252
PLT mutants	Fig 2E-F	401
Mitosis induction	Fig 3A-B	30 (3720 data points)
HTR2::CDT1-GFP	Fig 3C	69 (4329 data points)
CYCD2;1::CYCD2;1::GFP	Fig 3E&H	31 & 52 (676 data points)
CYCB::GFP	Fig 3D&G	190 & 27 (2619 data points)
KN::GFP-KN	Fig 3F&I	180 (479 data points)
LRC differentiation	Fig 4B, G	5 (250 data points)
Ep differentiation	Fig 4C,D,H	34 (415 data points)
Co differentiation	Fig 4E, I, S4A	85 (611 data points)
En differentiation	Fig 4F, J, S4B,C	68 (194 data points)
shr/scr mutants	Fig 5B, C	314
CYCD6;1::GFP	Fig 5D	109
SHR::SHR::GFP	Fig 5E, S5B	101
FEZ::FEZ::GFP	Fig 6B, S6A	47
SMB::SMB::GFP	Fig 6C, S6B	68
fez/smb mutants	Fig 6D	529
fez dynamics	Fig 6E, S6C	19 (1520 data points)
smb dynamics	Fig 6F, S6C	34 (2720 data points)
Multiple ablations	Fig S1C-F	90
Continued divisions	Fig S1G	35
Capsella rubella	Fig S1H	18
Nicotiana benthamiana	Fig S1I	9
Oryza sativa	Fig S1J	3
HU treatment	Fig S1K-M	15
Cell file collapse	Fig S1N	6
Competence	Fig S2A	439
cre triple	Fig S2B	158
BAP treatment	Fig S2C	172
EBL treatment	Fig S2D	161
LRC in *35S::PLT2-GR*	Fig S2E	3
PLT1::ECFP	Fig S2F	68
PLT2::YFP	Fig S2G	52
Cortex variation	Fig S3A-B	59 (6195 data points)

Division rate at 12h	Fig S3C	174
ERF115::NLS-GFP	Fig S3D-G	116
ERF115-SRDX	Fig S3H	294
WIND1 lines	Fig S3I	468
RBR1::RBR1::GFP	Fig S3J	124 (3000 data points)
WIND1::GFP	Fig S3K	45
PAT1::GFP	Fig S3L	43
WER::GFP outer division	Fig S4D, E	23
E4722 outer division/NAA	Fig S4F	97
SCR::SCR-YFP outer division/NAA	Fig S4G	61
WER::GFP outer division/NAA	Fig S4H, I	17
CYCD6;1::CYCD6;1::GFP	Fig S5A	45
SCR::SCR::YFP	Fig S5C	72
MC formation/GA-PAC	Fig S5E-G	191
fer-4	Fig S7A	69
FER::FER-GFP	Fig S7B	49
mslΔ5	Fig S7C	135
MSL9::GFP	Fig S7D	42
MSL10::GFP	Fig S7D	49
mca and *the1-3*	Fig S7E	378
trm6trm7trm8	Fig S7F	21
LHW::n3GFP	Fig S7G	28
TMO5::n3GFP	Fig S7G	32
LOG4::n3GFP	Fig S7G	41
TCSn::GFP	Fig S7G	28
